# Interaction of Follicle-Stimulating Hormone and Stem Cell Factor to Promote Primordial Follicle Assembly in the Chicken

**DOI:** 10.3389/fendo.2019.00091

**Published:** 2019-02-19

**Authors:** Changquan Guo, Guang Liu, Dan Zhao, Yuling Mi, Caiqiao Zhang, Jian Li

**Affiliations:** Department of Veterinary Medicine, College of Animal Sciences, Zhejiang University, Hangzhou, China

**Keywords:** primordial follicle, follicle-stimulating hormone, stem cell factor, folliculogenesis, chicken

## Abstract

Follicle-stimulating hormone (FSH) and KIT signaling are required for ovarian development. In this study the interactive effect of FSH and stem cell factor (SCF) on folliculogenesis was investigated in the chicken. Correlated changes between the FSH receptor and the expression of *KIT* signaling genes were seen to be involved in the formation of the chicken primordial follicles. Follicle-stimulating hormone and SCF displayed a reciprocal stimulating effect in the promotion of folliculogenesis involving elevated phosphorylation of mitogen-activated protein kinases (MAPK) and protein kinase B (AKT) proteins. Knockdown of *c-KIT* or *SCF* reduced the stimulatory effect of FSH on KIT signaling as well as upon MAPK and AKT phosphorylation. Treatment of FSH and SCF in combination enhanced ovarian cell proliferation and N-cadherin expression, but inhibited cell apoptosis and E-cadherin expression. Overall, the reciprocal stimulating effect of FSH and SCF in promoting chicken follicle assembly involving accelerated ovarian cell proliferation, N-cadherin expression, inhibited cell apoptosis, and E-cadherin expression is demonstrated.

## Introduction

The reproductive lifespan of female mammals is determined at the time of birth through establishment of a primordial follicle pool in which the oocytes are enclosed by a layer of flattened pre-granulosa cells (1, 2). During the formation of primordial follicles, germ cell cysts break apart into single oocytes surrounded by pre-granulosa cells, this process may be organized by some stimulus other than apoptosis such as migration of pre-granulosa cells to envelop the oocyte (3). Germ cells undergo mitosis or meiosis in chicken embryos predominantly under the control of gonadotropins, and there are few germ cells to continue proliferation in the chicken ovarian cortex after hatching (4). In the hatched chicken, the initial development of the primordial follicle pool occurs during the first 4 days post-hatching through germline nest breakdown and the enclosure of oocytes with pre-granulosa cells in the chicken. However, the basic mechanisms regulating these processes in chicken ovaries remain poorly understood. Studies of mammalian females have shown that they are incapable of producing oocytes and follicles after birth (5, 6). In this respect, the oocytes in the primordial follicles represent the entire available reproductive resource.

The role of hormones, including gonadotropins, estradiol (E_2_), and progesterone, that act during the formation of primordial follicles has been explored in various mammalian species (7–9). Follicle-stimulating hormone (FSH) has multiple effects on ovary development in mammalian such as folliculogenesis and survival of preantral follicles (10). The inhibitory effect of the anti-FSH antibody and the stimulatory effect of FSH has indicated that FSH acts to accelerate primordial follicle formation in hamster ovaries where the FSH receptor (FSHR) as regulated by the FSH and E_2_ accounted for primordial follicle formation (11, 12). FSH was also seen to promote primordial follicle formation by stimulating local activin signaling pathways and the expression of oocyte-specific transcription factors in the mouse (13). In this way, there is abundant evidence to demonstrate that FSH can promote the primordial follicle assembly in mammalian ovaries. However, the effect of FSH on chicken primordial follicle assembly remains largely unclear.

During follicular development FSH regulates both the release of several paracrine factors and the function of the oocyte-granulosa cells interactions. Wang and Roy showed that the action of FSH involved in folliculogenesis seems to occur via a local factor stem cell factor (SCF) (11). Stem cell factor is also known as KIT ligand (KITL) and its receptor KIT represents the origin of the KIT signaling that is known for its function to promote cell survival, proliferation, and differentiation. Moreover, KIT signaling drives oocyte growth in preantral follicles by changing the relationships between oocyte size and follicle size (14). KIT autophosphorylation is able to activate several downstream cascades, including phospha-tidylinositol3-kinase (PI3K), and mitogen-activated protein kinase (MAPK) pathways (15, 16). Interactions between FSH and the PI3K and MAPK pathways are also crucial for the development of ovarian cells (17). However, whether FSH is able to cooperate with KIT signaling in chicken ovarian folliculogenesis remains unknown.

There are lots of signaling participating in primordial follicles formation (18, 19). Furthermore, development of primordial follicles is under the strict interactive regulation of hormones, cytokines, connexin, and transcription factors (20, 21). FSH could regulate cell adhesive molecules and growth factors to modulate the formation of primordial follicles (11, 22). However, it needs to be clarified whether FSH can interact with KIT signaling to regulate avian primordial follicle development.

Since folliculogenesis is pivotal for future laying performance in poultry species, we hypothesized that FSH could promote primordial follicle assembly through endogenous KIT signaling. Through *in vivo* FSH administration and *in vitro* ovarian tissue culture, we investigated the putative reciprocal effect of FSH and SCF/KIT signaling to promote follicle development in the early stages of chicken development.

## Materials and Methods

### Treatments of Animals and Tissue Collection

Fertilized Hyline chicken (*Gallus gallus*) eggs were incubated in an egg incubator at 38.5°C, 60% humidity with a 12 h light and 12 h darkness cycle. After hatching the chicks were raised under same photoperiod cycles. All procedures were performed in accordance with the *Guiding Principles for the Care and Use of Laboratory Animals of Zhejiang University*. The female chicks were injected intraperitoneally with 5 IU FSH [human FSH-Fc/Fc heterodimer, KN015, (23)] in 0.1 ml of PBS solution at Day 4. The control chicks received 0.1 ml of PBS solution. On Day 6 the chicks were sacrificed after anesthesia and the left ovaries were collected for morphological and biochemical analyses.

### Organ Culture

Individual left ovaries of the 4-day-old chicks were cut into 2 to 4 pieces for organ culture. The ovarian fragments were cultured onto 0.45 μm Millipore membrane filters. Each ovarian fragment was cultured in six-well plates at 38.5°C, 5% CO_2_ with 2 ml Dulbecco's Modified Eagle's Medium–Ham's F-12 medium (DMEM/F-12, 1:1, Hyclone, Utah, USA) supplemented with 2 mM glutamine, 100 IU/mL penicillin, and 100 μg/mL streptomycin (Gibco), 10 μg/ml insulin, 5 μg/ml transferrin, 30 nM selenite (ITS medium, Sigma Aldrich, St. Louis, MO, USA), 5% fetal calf serum (FCS, Hyclone, Utah, USA) as the complete culture medium. Culture medium and treatments were renewed every 36 h. The ovaries were treated with 0.01 IU/ml FSH, 20 ng/ml SCF (78062, Stem Cell Inc, Canada), and FSH+SCF, respectively. For the 5-bromo-20-deoxyuridine (BrdU) incorporation assay ovaries were incubated with the complete medium supplemented with 0.1 mg/ml BrdU (Sigma, Aldrich, USA) at 38.5^o^C, 5% CO_2_ for 12 h. The ovarian fragments were fixed in 4% paraformaldehyde for morphological observation after 72 h of culture. After 48 h culture the ovarian fragments were collected for Western blot and qRT-PCR analysis.

### Culture of Ovarian Cells

Ovaries from Day 4 chicks were cut into small fragments and digested with 1 mg/ml trypsin-EDTA (Solarbio, Beijing, China) (24). The dispersed cells were filtered through a 50 μm mesh and then seeded in collagen-treated six-well plates supplemented with ITS and 5% FCS, at a density of 5 × 10^5^ in 2 ml/well of DMEM/F-12 (Hyclone, Utah, USA). Ovarian cells were treated with 0.01 IU/ml FSH and incubated at 38.5°C and 5% CO_2_.

### Transfection of *SCF* and *c-KIT* siRNAs

At 70% confluence of the cultured ovarian cells, the cells after FSH treatment were transfected for 24 h with either a SMART pool of small interfering RNA (siRNA) specific for SCF, c-KIT, or a non-silencing control (GenePharma Co., Ltd., Shanghai, China) using Lipofectamine 2,000 (Invitrogen, California, USA) in accordance to the manufacturer's instructions. After 24 h, the transfection mixtures were replaced with regular medium. The antisense sequences of primers for siRNAs are listed in Table 1.

**Table 1:** Antisense primers for siRNA.

**Gene**	**Antisense primer sequences(5′-3′)**
*Non-silencing control*	ACGUGACACGUUCGGAGAATT
*siSCF-chicken-506*	AUUGAUUAUCCUUGUGAGGTT
*siSCF-chicken-799*	UUACUGCUAGUAUUACUGCTT
*Sic-KIT-chicken-870*	UUAGCUAGUGCUUUCAAGGTT
*Sic-KIT-chicken-1158*	UUAAAUGUUACAGAGGAGCTT

### Morphological Observation

The ovaries were cut into 5 μm paraffin sections and processed for histological observation. Five serial sections across the largest cross-section of the ovaries were selected for staining with hematoxylin and eosin. The follicles per cortical area (mm^2^) at each developmental stage were counted in these sections. The oocyte nuclei had to be visible in the follicle in order to be counted. Follicles were classified as in either primordial or growing stages. The primordial follicles consist of an oocyte partially or completely encapsulated by flattened squamous pregranulosa cells whereas growing follicles contain one or more cuboidal granulosa cells in successive layers around the oocytes.

### Immunohistochemistry

The chicken ovarian sections were deparaffinized and hydrated. Treatment with 3% H2O2 for 20 min to quench endogenous peroxidase activity was followed by antigen retrieval which was performed for 10 min in a 10 mM citrate buffer (pH 6.0). The sections were washed three times with PBS and then blocked for 20 min with 5% normal goat serum. Slides were incubated with primary antibodies of rabbit anti-c-KIT (1:100), rabbit anti-CDK2 (1:200), rabbit anti-CCND1 (1:200), rabbit anti-E-cadherin (1:100, Boster, Wuhan, China), rabbit anti-N-cadherin (1:200, Novus Biologicals, Littleton, USA), and rabbit anti-caspase-3 (1:100, Abcam, Cambridge, UK) overnight at 4°C. Subsequently the sections were incubated with goat anti-mouse or goat anti-rabbit secondary antibody (1:500, Abcam, Cambridge, UK) for 60 min at 37°C. Finally, immunostaining was visualized using three, 3-diaminobenzidine (Boster Bioengineering Co., Ltd., Wuhan, China) and hematoxylin counterstaining. Positive staining (dark brown) in each section was examined in at least five random fields at 400 × magnification. A negative control was prepared in an identical manner except that the primary antibody had been replaced with normal serum, and five slides across maximal cross section were used.

### Immunofluorescence Staining

The ovarian sections were incubated with rabbit anti-FSHR (1:500, ABclonal technology Inc, Wuhan) for 12 h at 4°C. The first antibody was verified by Western blot analysis. After incubation with the secondary antibody at 37°C for 1 h, the sections were counterstained with 4′, 6-diamidino-2-phenyl indole (DAPI, Sigma Aldrich, St. Louis). The negative sections were incubated with normal serum instead of primary antibody, and five slides across maximal cross section were used. Mounted slides were visualized using a laser scanning confocal microscope (FV1000, Olympus, Tokyo, Japan).

### BrdU Incorporation

The ovarian fragments for the BrdU incorporation assay were fixed in 4% paraformaldehyde. Paraffin sections were then deparaffinized and hydrated, followed by 2 M HCl denaturation for 30 min at room temperature, and neutralization by 0.1 M sodium tetraborate for 10 min. The slides were then incubated with mouse anti-BrdU polyclonal antibody (1:200, G3G4, Abcam, Cambridge, UK) as the primary antibody and subsequently with TRITC-labeled goat anti-mouse IgG as the secondary antibody. Sections were then counterstained with DAPI and mounted slides were visualized using a confocal microscope (FV1000, Olympus, Tokyo, Japan). The number of BrdU positive and total ovarian cells was counted in the maximal cross section and expressed as a percentage of BrdU labeled cells to the total number of ovarian cells (BrdU index).

### TUNEL Assay

The ovarian sections were incubated with the reagents using a terminal deoxynucleotidyl transferase-mediated deoxyuridine triphosphate nick-end labeling kit (TUNEL, Vazyme, Nanjing) according to the manufacturer's instruction. Slides were visualized using a confocal microscope (FV1000, Olympus, Tokyo, Japan) and the number of TUNEL positive and total ovarian cells was counted in a maximal cross section and expressed as a percentage of the TUNEL labeling ovarian cells to the total number of ovarian cells.

### *In situ* Hybridization

Specific primers used for production of the digoxigenin-labeled probes to detect *SCF* mRNA were: 5′-AATCTCCCAAATGATTATCTGATAACCCTTAAATA-3′ and 5′-GATAAGAACGACTGCATTATGCCTTCAACTGTAGA-3′. For *in situ* hybridization, tissue sections (6 μm thickness) were deparaffinized and permeabilized with 4 mg/ml pepsin diluted by 3% citrate for 2 min at 37°C, followed by incubation with the hybridization buffer containing digoxigenin-labeled probes for 12 h. The sections were then developed as described in the manufacturer's protocol (Boster Bioengineering Co., Ltd., Wuhan, China) and counterstained with haematoxylin. The negative control was prepared in an identical manner except that the primary antibody was replaced with normal serum.

### RNA Extraction and qRT-PCR

Total RNA was extracted from the ovaries or cells using a Trizol reagent (Invitrogen Co., Carlsbad, CA, USA). The cDNA was generated with 2 μg total RNA by using a SuperScript First-Strand Synthesis System (Fermantas, Glen Burnie, MD) based upon the manufacturer's protocol. Quantitative real time PCR (qPCR) was used to assess the expression of *FSHR, SCF/KITL, c-KIT*, Newborn ovary homeobox gene (*Nobox*), TBP-associated Factor 4b (*TAF4b*). qPCR was carried out in triplicate using a SYBR Premix Ex Taq™ (TaKaRa Bio Inc., Japan) in a CFX96 real-time PCR detection system (Bio-Rad, USA). After normalization with glyceraldehyde-3-phosphate dehydrogenase (*GAPDH*), relative mRNA expression levels in the samples were calculated using the comparative threshold cycle method. The delta-delta CT method was used to calculate relative fold-change values between samples (4). The sequences of the primers for PCR analysis are listed in Table 2.

**Table 2:** Primers for PCR analysis.

**Gene**	**Accession no**.	**Primer sequences (5′-3′)**	**Product length/bp**
*c-KIT*	NM.204361.1	GTTGAAACCAAGCGCCCATT	195
		AATGAATCTCGCTTCCGCCT	
*KITL/SCF*	NM_205130.1	TCTTATGGCATGTTTAGCTTTTGA	139
		TGCAAACTCCTTGTAGACCTCA	
*FSHR*	NM.205079.1	ACCTGCCTGGATGAGCTAAA	136
		ATCCATGACTTGGCAGGAAG	
*GADPH*	M.11213.1	ACTGTCAAGGCTGAGAACGG	204
		AGCTGAGGGAGCTGAGATGA	
*TAF4b*	XM.419170.5	TGGATCTGGGACAGAGGGTT	113
		AGTCCCTGAGACAGACACGA	
*Nobox*	XM.417224	CAGAATCGCAGGGCAAAG	143
		TGAGGCAATGGAGGCACT	

### Western Blot Analysis

The left ovaries were homogenized using a RIPA lysis buffer and 1 mM PMSF (Beyotime, Nanjing, China). Proteins were separated on a 10% SDS-polyacrylamide gel electrophoresis and transferred onto a PVDF membrane. The membrane was incubated in 5% dried skimmed milk at room temperature for 1 h and subsequently incubated overnight at 4°C with corresponding primary antibodies including mouse anti-PCNA (1:500, Abcam, Cambridge, UK), rabbit anti-c-KIT (1:100, Boster, Wuhan, China), rabbit anti-FSHR (1:500, ABclonal technology, Wuhan), rabbit anti-Akt and phospho-Akt (1:200, Cell Signaling Technology, Massachusetts, USA), rabbit anti-MAPK1 (1:100, Boster, Wuhan, China) and phospho-MAPK1/3 (1:500, Novus Biologicals, Littleton, USA), rabbit anti-CDK2 (1:200, Boster, Wuhan, China), rabbit anti-CCND1 (1:200, Boster, Wuhan, China), rabbit anti-E-cadherin (1:100, Boster, Wuhan, China), rabbit anti-N-cadherin (1:200, Novus Biologicals, Littleton, USA), rabbit anti-caspase-3 (1:100, Abcam, Cambridge, UK), and rabbit anti-β-catenin (1:200, DSHB, Iowa City, USA), respectively. The membranes were then incubated at 37°C with respective secondary antibodies for 60 min. The β-actin bands were used as an internal control. Negative control of FSHR was prepared in an identical manner except that the primary antibody was replaced with PBS. For protein quantification, the bands in the images were digitized, and analyzed using ImageJ software (developed by the National Institutes of Health, http://rsbweb.nih.gov/ij/).

### Statistical Analysis

All data were expressed as the means ± SEM and analyzed using the SPSS 21.0 software *t*-test or One-way analysis of variance (ANOVA) with Duncan's multiple-range tests. Graphs were drawn in GraphPad 6.0 and *P* < 0.05 was considered as a statistically significant difference.

## Results

### Expression of *FSHR, SCF* and *c-KIT* mRNAs in the Developing Chicken Ovaries

To determine correlated changes in the expression of *FSH, SCF*, and *c-KIT* mRNAs during the crucial period of germline cyst breakdown and primordial follicle formation, the cellular localizations, and expression patterns of these genes was determined using immunofluorescence, *in situ* hybridization, immunohistochemistry, qRT-PCR, and Western blot.

**Figure 1:**
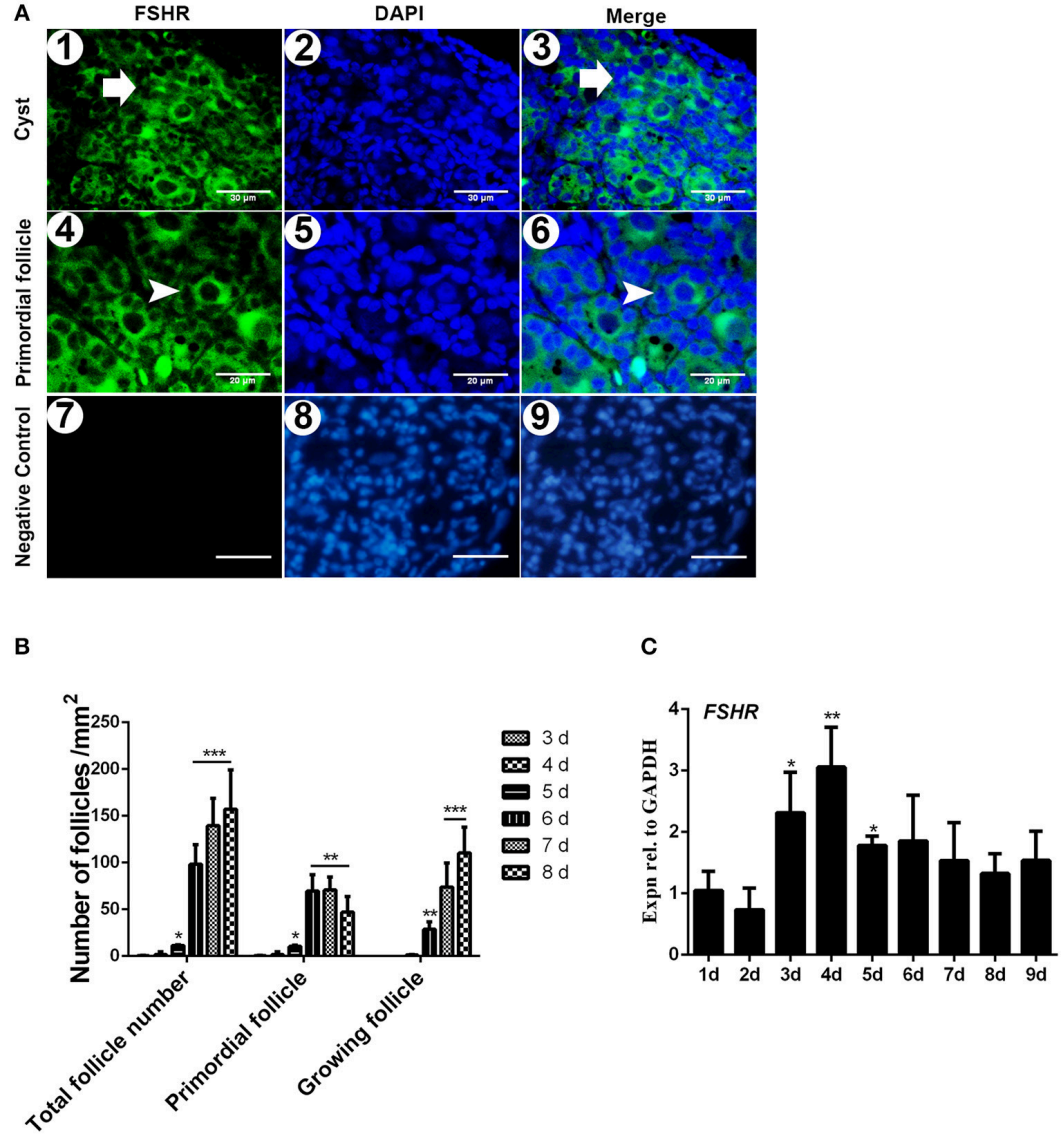
Expression of FSHR mRNA increased significantly on the initial formation of chicken primordial follicles. **(A)** Cellular localization of FSHR in 6-day-old chicken ovaries. FSHR (green) existed in both the cysts (arrows in 1–3) and primordial follicles (arrowheads in 4 and 6). Scale bars (1–3): 30 μm. Scale bar (4–6): 20 μm. Negative control scale bars (7–9): 30 μm. DAPI (blue, 2 and 5) was used to identify the nuclear DNA. **(B)** Changes of total, primordial, and growing follicle number from Day 3 to 8. **(C)** *FSHR* mRNA expression was measured by qRT-PCR in ovaries from the first to 9th day in the chickens. GAPDH was used as a normalization control. *T*-tests were used to determine statistically significant differences. The values are the mean ± SEM of six experiments. Asterisks indicate significant differences (*P < 0.05, **P < 0.01, ***P < 0.001).

**Figure 2:**
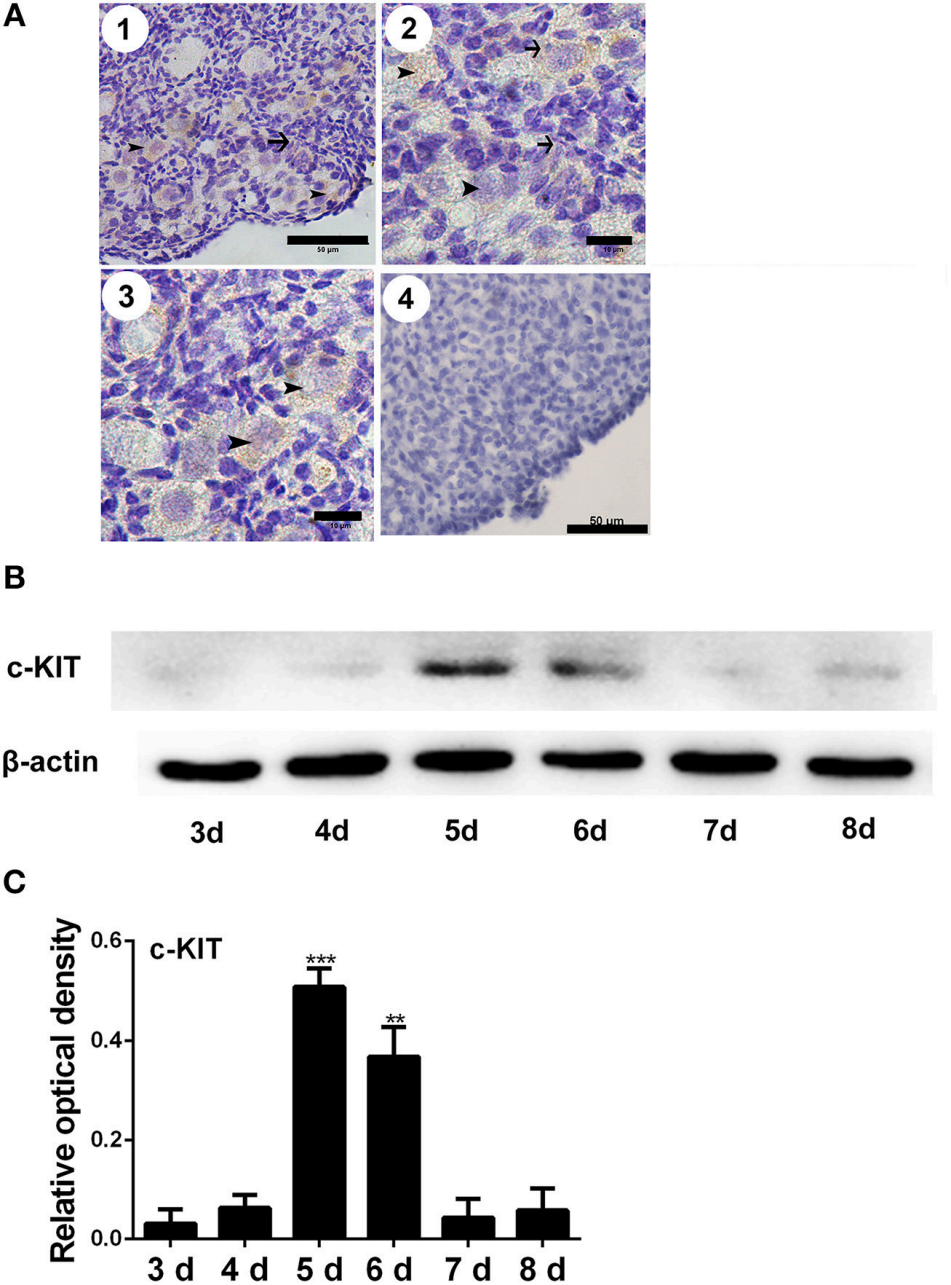
Changes of c-KIT during primordial follicle formation. Brown staining indicates c-KIT, arrows, and arrowheads represent the somatic cells and oocytes, respectively, in 6-day-old chicken ovaries (**A**-1, scale bar: 50 μm). An enlarged picture is also included (**A**-2 and 3, scale bar: 10 μm). A negative control (**A**-4, scale bar: 50 μm). **(B)** Expression of c-KIT by Western blot in 3 to 8-day-old chicken ovaries, normalized to β-actin. **(C)** Gray analysis indicates that c-KIT had increased significantly from days 4 to 6. *T*-tests were used to determine statistically significant differences. The values are the mean ± SEM of six experiments. Asterisks indicate significant differences (**P < 0.01, ***P < 0.001).

**Figure 3:**
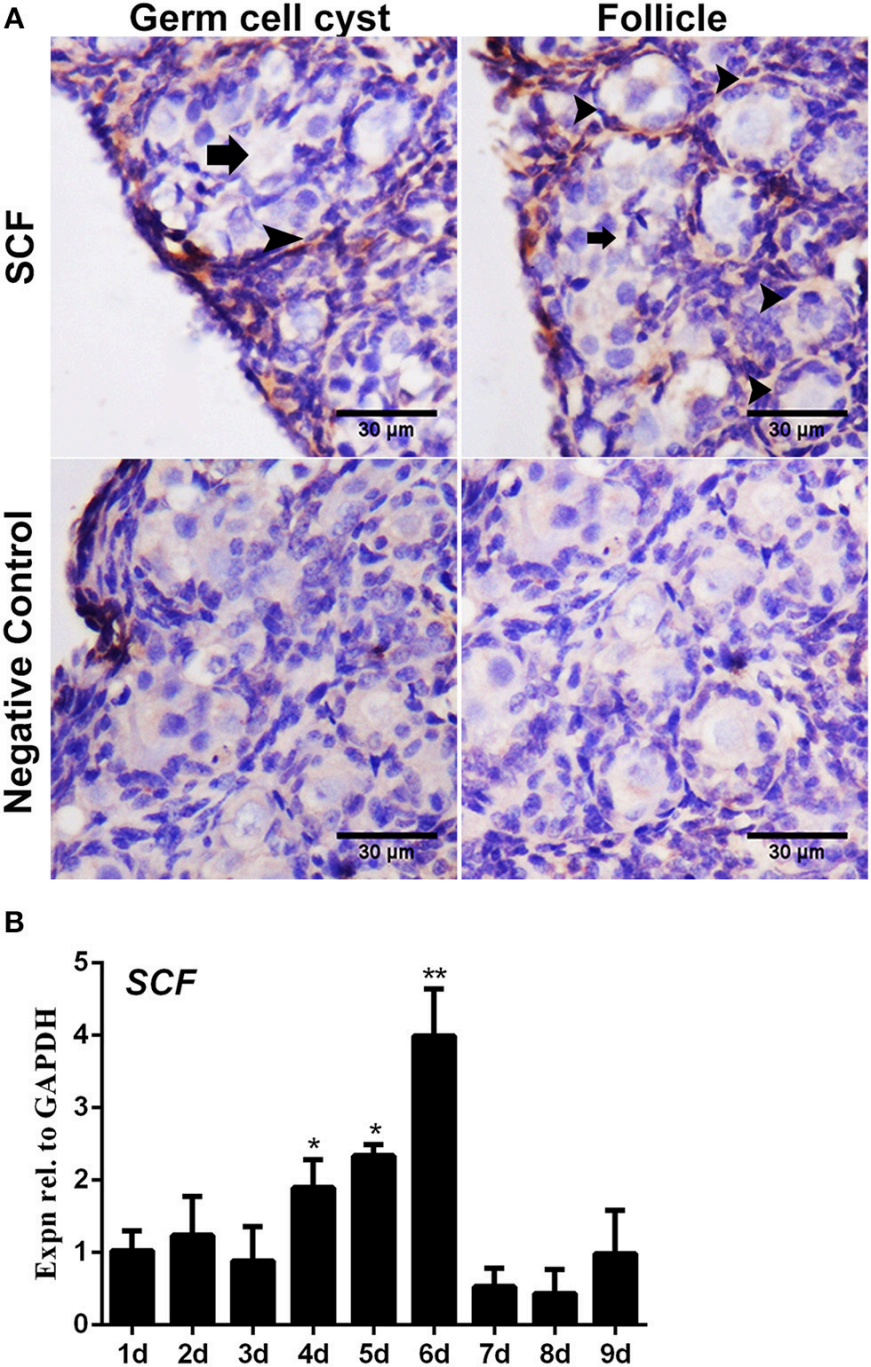
Expression of *SCF* mRNA in the chicken ovaries. SCF mRNA showed staining in the cell cytoplasm by *in situ* hybridization. **(A)** *SCF* mRNA was detected in the cytoplasm in 6-day-old chicken ovarian cells. Arrows and arrowheads represent the cysts and follicles, respectively. **(B)** *SCF* mRNA expression was measured by qRT-PCR in the chicken ovaries from Day 1 to 9. GAPDH was used as the normalization control. *T*-tests were used to determine statistically significant differences. The values are the mean ± SEM of six experiments. Asterisks indicate significant differences (*P < 0.05, **P < 0.01).

The immunostaining of FSHR was located on both the germ cells cysts and primordial follicles in the 6-day-old chicken ovaries (Figure 1A). From Day 6 the number of total follicles, primordial follicles, and growing follicles began to increase by 760.5%, 572.8%, and 2579.9% (Figure 1B, ^*^P < 0.05, ^**^P < 0.01, ^***^P < 0.001). From Day 2 to Day 3 the abundance of *FSHR* mRNA increased significantly and remained at a high level on Day 4 in chicken ovaries, increased by 191.4% (Figure 1C, ^*^P < 0.05, ^**^P < 0.01). This sharp increase of *FSHR* mRNA appeared prior to the extensive formation of the primordial follicles which occurs from Day 6 onwards. Meanwhile, immunostaining of c-KIT was detected on oocytes in cysts, primordial follicles, and on their surrounding somatic cells (Figure 2A). The c-KIT protein increased significantly by 709.3% from Day 4 to Day 5 remaining at a high level on Day 6, then decreased sharply thereafter in the ovaries (Figures 2B,C). Meanwhile, *SCF* mRNA was located on the surrounding somatic cells of the germ cell cysts and follicles in the chicken ovarian cortex (Figure 3A). The expression of *SCF* mRNA in the chicken ovaries increased significantly by 114.4% from Day 3 to Day 4, further increasing by 289.6% to its peak level on Day 6, then decreasing to a much lower level from Day 7 till Day 9 (Figure 3B, ^*^P < 0.05, ^**^P < 0.01).

### FSH Enhanced Primordial Follicle Formation and KIT Signaling *in vivo*

**Figure 4:**
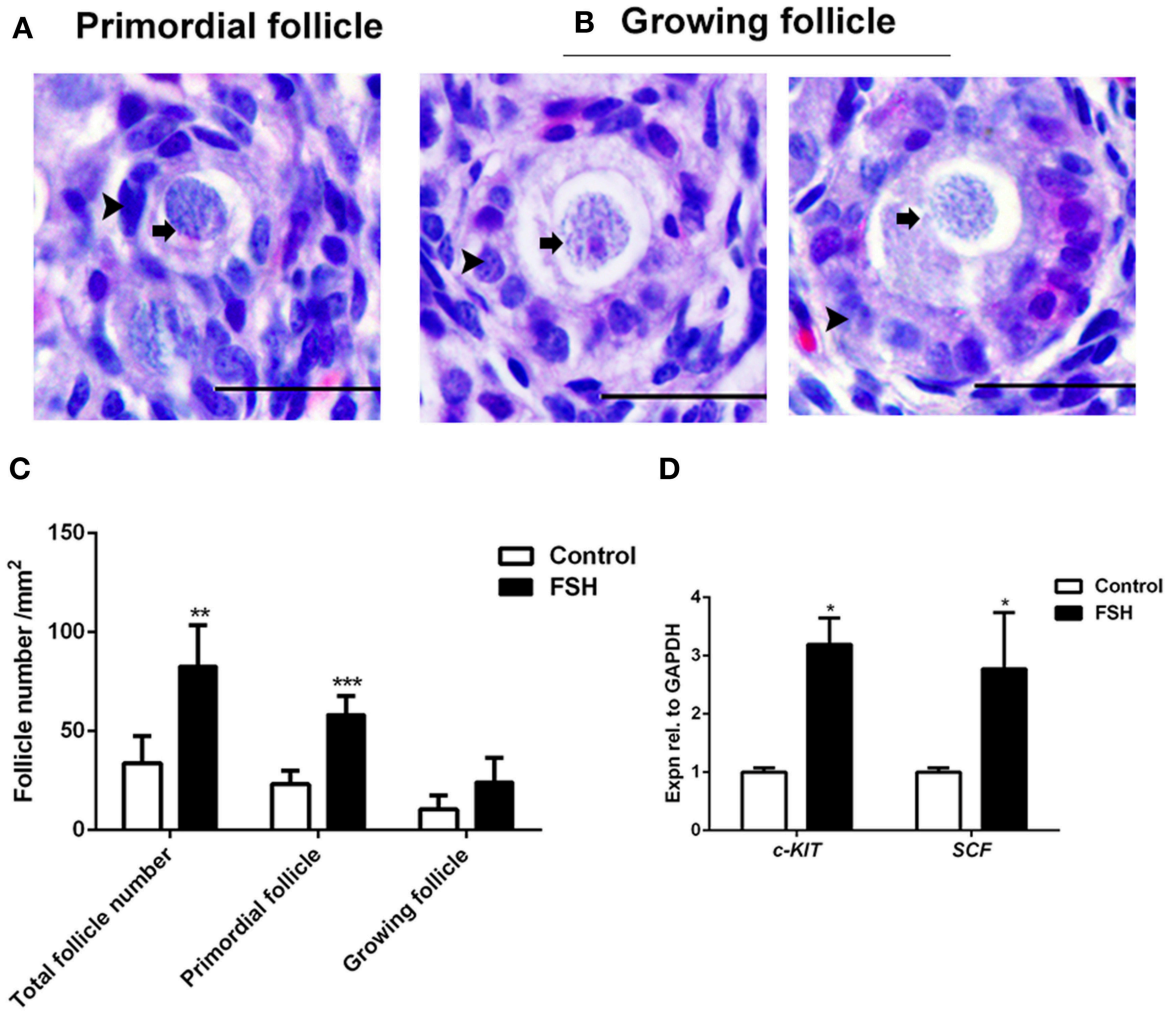
Effects of FSH treatment on chicken folliculogenesis *in vivo*. **(A,B)** Morphology of the primordial and growing follicles in 6-day-old chicken ovaries. Scale bar: 20 μm. Arrowheads and arrows represent the somatic cells and oocytes, respectively. **(C)** Changes in the primordial and growing follicle numbers after FSH treatment. **(D)** The *c-KIT* and *SCF* mRNA expressions were measured by qRT-PCR in ovaries from the 6-day-old chickens after FSH treatment at day 4. *GAPDH* was used as the normalization control. *T*-tests were used to determine statistically significant differences. The values are the mean ± SEM of six experiments. Asterisks indicate significant differences (*P < 0.05, **P < 0.01, ***P < 0.001).

**Figure 5:**
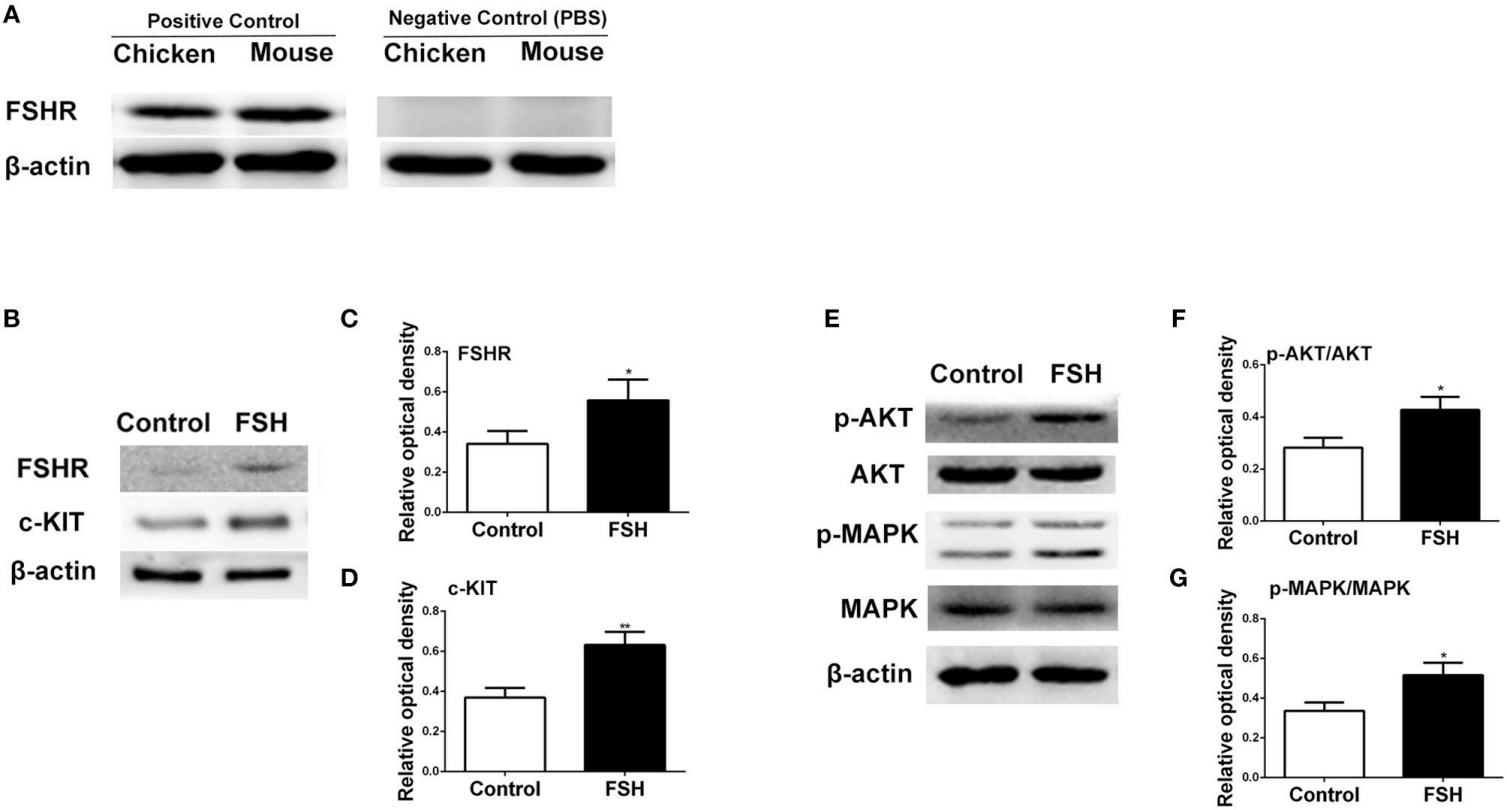
Effects of FSH treatment on KIT signaling *in vivo*. **(A)** Western blot and gray analysis of FSHR positive and negative controls in the 6-day-old chicken and mouse ovaries. Western blot and gray analysis indicate that FSH significantly promoted expression of FSHR **(B,C)**, c-KIT **(B,D)**, p-AKT/AKT **(E,F)**, p-MAPK/MAPK **(E,G)**. *T*-tests were used to determine statistically significant differences. The values are the mean ± SEM of six experiments. Asterisks indicate significant differences (*P < 0.05, **P < 0.01).

Since the expression of *FSHR* mRNA significantly increased during the process of the primordial follicle assembly, we treated the chickens with FSH *in viv*o and found that the number of primordial follicles increased significantly after FSH administration. This resulted in an increase of the number of total follicles and primordial follicles by 146.2% and 152.8% (Figures 4A–C, ^*^P < 0.05, ^**^P < 0.01, ^***^P < 0.001). Meanwhile, treatment with FSH significantly increased the expression of both *SCF* and *c-KIT* mRNAs in the chicken ovaries by 176.5 and 218.8% (Figure 4D, ^*^P < 0.05, ^**^P < 0.01, ^***^P < 0.001). We verified the cross reactivity of FSHR antibody with chicken and mouse ovarian FSHR by Western blot analysis, including the FSHR positive and negative controls (Figure 5A). Treatment of FSH displayed a marked up-regulation of its own receptor (Figures 5B,C, ^*^P < 0.05). The c-KIT protein level was increased significantly by 77.1% after FSH treatment (Figures 5B,D, ^*^P < 0.05, ^**^P < 0.01), where FSH also enhanced the ratio of p-AKT/AKT by 56.9% (Figures 5E,F, ^*^P < 0.05). Moreover, FSH enhanced the ratio of p-MAPK/MAPK by 47.4% (Figures 5E,G, ^*^P < 0.05).

### SCF Enhanced the Stimulatory Effect of FSH on FSHR and KIT Signaling Components

**Figure 6:**
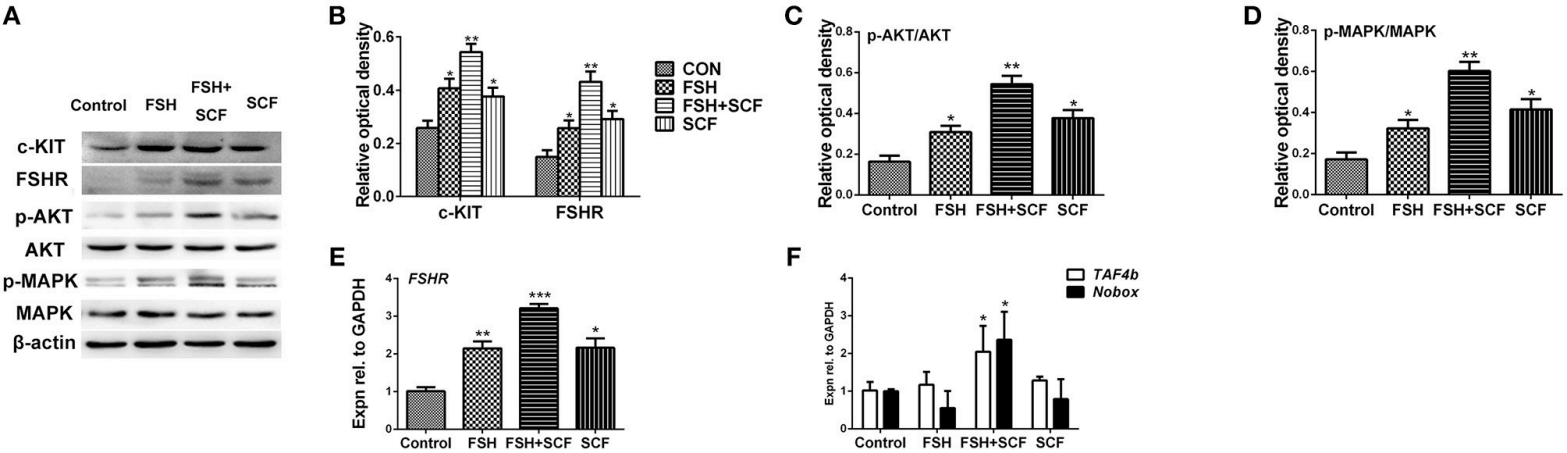
SCF enhanced the stimulatory effect of FSH *in vitro*. The 4-day-old chicken ovaries were treated with FSH and SCF for 2 days in culture. **(A,B)** Western blot and gray analysis indicate that SCF enhanced the effect of FSH on promoting c-KIT and FSHR protein expressions. **(E)** qRT-PCR analysis indicates that SCF could enhance the effect of FSH to promote FSHR mRNA expression. Western blot and gray analysis indicate that SCF could enhance the effect of FSH to increase ratio of p-AKT/AKT **(A,C)** and ratio of p-MAPK/MAPK **(A,D)**. **(F)** FSH also combined with SCF to promote *TAF4b* and *Nobox* mRNA expression. *T*-tests were used to determine statistically significant differences. The values are the mean ± SEM of six experiments. Asterisks indicate significant differences (*P < 0.05, **P < 0.01, ***P < 0.001).

To confirm the reciprocal effect of FSH and KIT signaling involved in follicle formation, cultured ovarian fragments were treated with SCF, and FSH alone or in combination. Results showed that either FSH or SCF treatment increased the expression of c-KIT protein. Together, FSH and SCF displayed a reciprocal effect on elevating c-KIT expression by 99.1% (Figures 6A,B, ^*^P < 0.05, ^**^P < 0.01). FSH and SCF also manifested a similar reciprocal effect on both FSHR protein and mRNA levels, leading to increased expression of both of these after combined treatment by 189.3 and 217.7% (Figures 6A,B,E, ^*^P < 0.05, ^**^P < 0.01, ^***^P < 0.001). FSH and SCF remarkably enhanced the ratios of p-AKT/AKT by 89.0–233.2%, p-MAPK/MAPK by 88.1–250.8%, alone or in combination (Figures 6A,C,D, ^*^P < 0.05, ^**^P < 0.01).

Although treatment of the cultured ovaries with FSH or SCF alone resulted in no detectable differences in the expressions of *TAF4b* and *Nobox* mRNAs, the combined treatment of FSH with SCF increased the expression of these two genes significantly by 101.0 and 136.4% (Figure 6F, ^*^P < 0.05).

### Knockdown of *c-KIT* and *SCF* mRNAs Weakened the Stimulatory Effect of FSH to KIT Signaling

**Figure 7:**
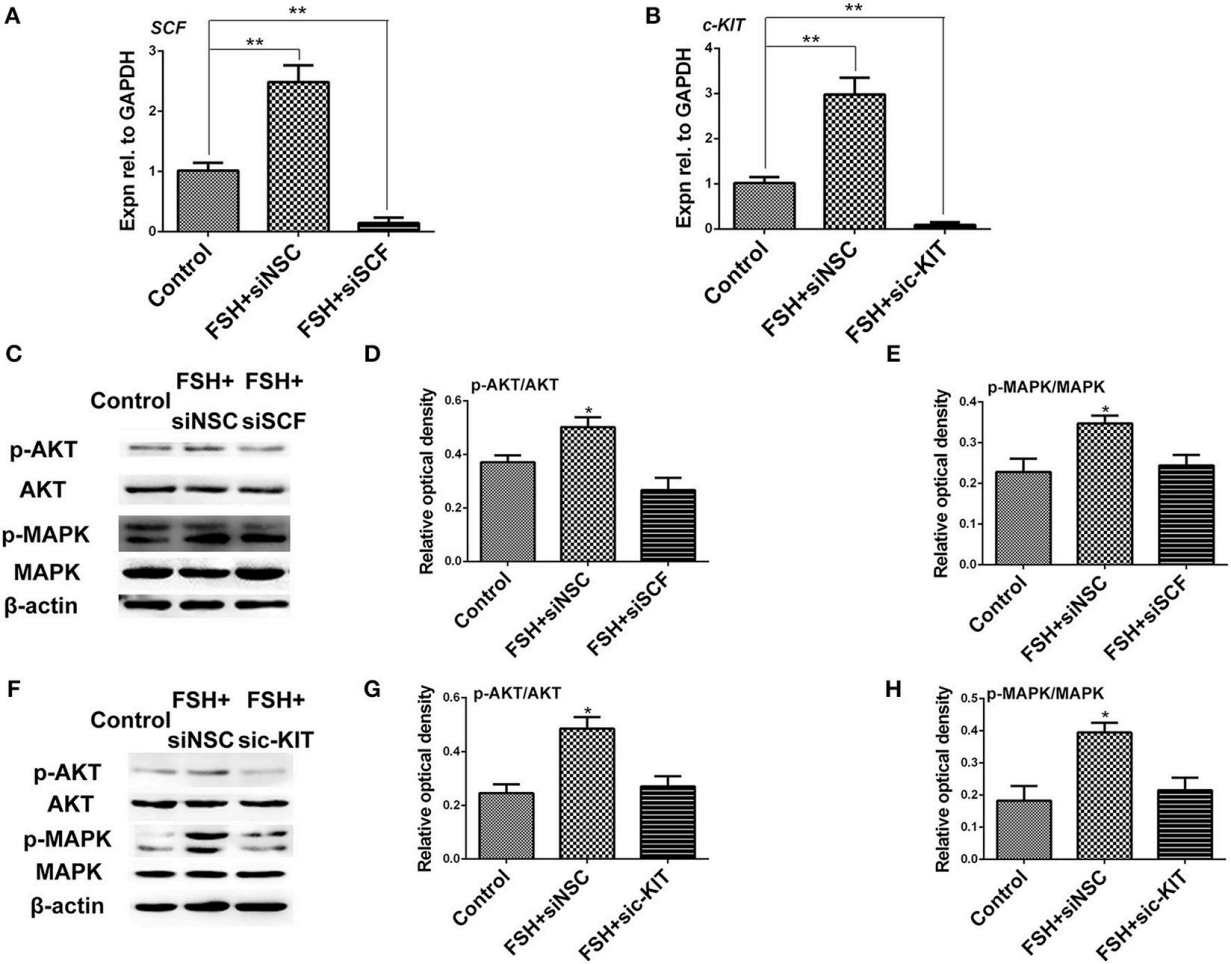
Knockdown of *SCF* and *c-KIT* mRNAs reduced the stimulatory effect of FSH on KIT signaling. **(A,B)** The ovarian cells were transfected for 24 h with siSCF or sic-KIT. qRT-PCR analysis indicates that siSCF or sic-KIT reduced the stimulatory effect of FSH on expression of *SCF* or *c-KIT* mRNAs. **(C–E)** Western blot and gray analysis indicate that siSCF reduced the stimulatory effect of FSH on ratio of p-AKT/AKT and p-MAPK/MAPK. **(F–H)** Treatment of sic-KIT reduced the stimulatory effect of FSH on ratio of p-AKT/AKT and p-MAPK/MAPK. “siNSC” stands for non-silencing control. *T*-tests were used to determine statistically significant differences. The values are the mean ± SEM of six experiments. Asterisks indicate significant differences (*P < 0.05, **P < 0.01).

To verify the interactive effect of FSH and KIT signaling on the process of the primordial follicle assembly, SCF, and c-KIT siRNA were individually designed to target and knockdown SCF and c-KIT expression in the chicken ovarian cells. The cultured ovarian cells were treated with FSH, and this was followed by siRNA transfection for 24 h. The results showed that FSH-stimulated increases in *SCF* or *c-KIT* mRNA expressions were significantly decreased by 94.1 and 97.0% by knockdown of either *SCF* or *c-KIT* mRNA (Figures 7A,B, ^*^P < 0.05, ^**^P < 0.01). The ratios of p-AKT/AKT, p-MAPK/MAPK were all decreased by knockdown of SCF by 46.9 and 29.7% (Figures 7C–E, ^*^P < 0.05) as well as c-KIT by 44.3 and 45.4% (Figures 7F–H, ^*^P < 0.05).

### FSH Increased Ovarian Cell Proliferation and N-Cadherin Protein *in vivo*

**Figure 8:**
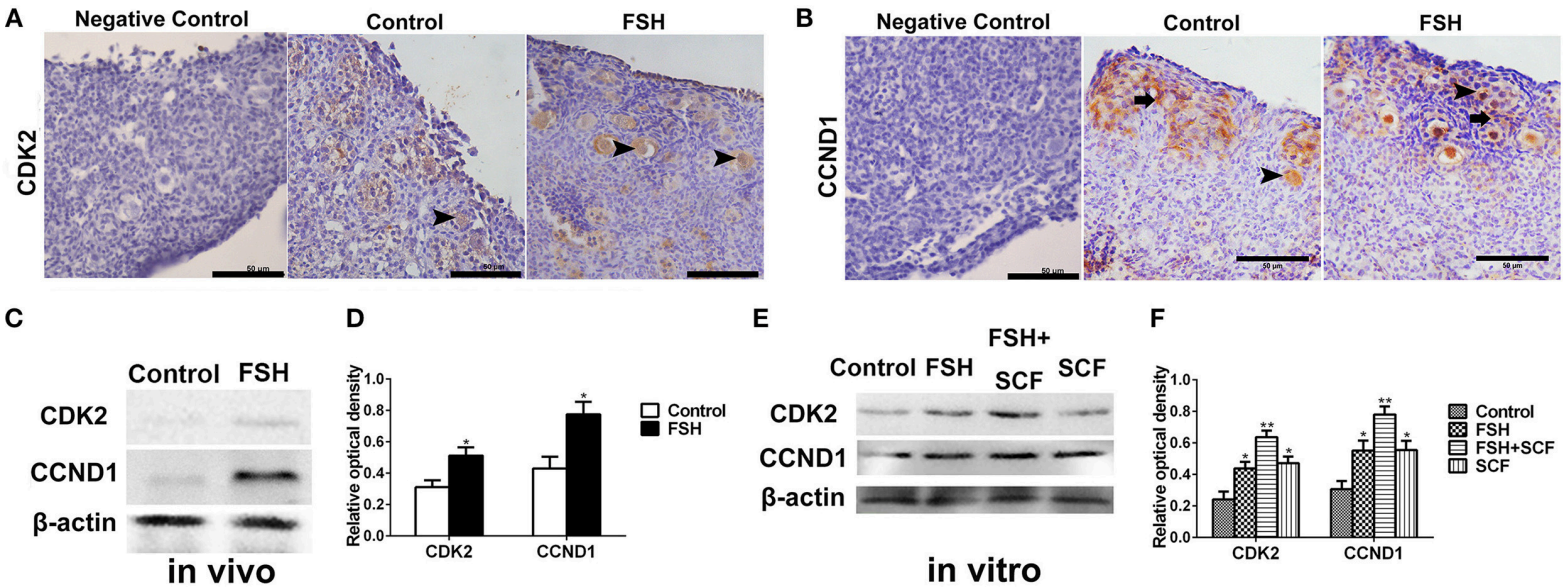
Combined effects of FSH and SCF on the expressions of cell cycling proteins CCND1 and CDK2. **(A–D)** 4-day-old chickens were treated by FSH *in vivo*. Brown staining represents the immunohistochemical antigen. **(E,F)** 4-day-old chicken ovaries were treated by FSH or SCF for 2 days *in vitro*. **(A)** Arrowheads represent oocytes stained with CDK2 antibody. **(B)** Arrowheads and arrows represent oocytes and somatic cells stained with CCND1 antibody. **(C,D)** Western blot and gray analysis indicate that FSH promoted the CDK2 and CCND1 protein expression *in vivo*. **(E,F)** Western blot and gray analysis indicate that SCF enhanced the effect of FSH on promoting the CDK2 and CCND1 protein expression *in vitro*. *T*-tests were used to determine statistically significant differences. The values are the mean ± SEM of six experiments. Asterisks indicate significant differences (*P < 0.05, **P < 0.01). Scale bars: 50 μm.

**Figure 9:**
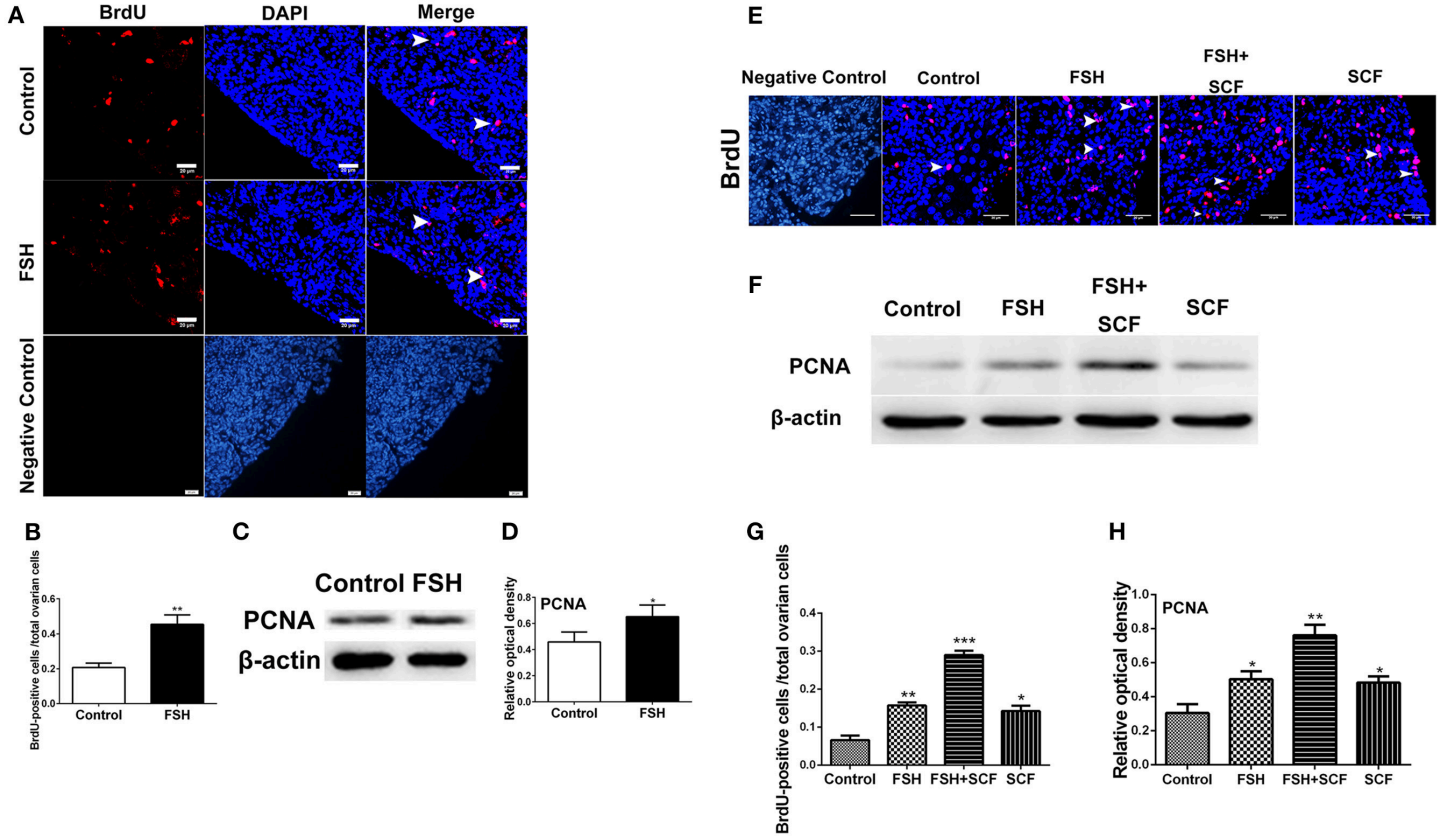
Combined effects of FSH and SCF on cell proliferation. **(A)** The 6th day chicken ovaries were incubated for 6 h with BrdU after treatment by FSH *in vivo*. Arrowheads represent ovarian cells with BrdU (red) marker in the ovarian cortex. DAPI (blue) represents the nuclear DNA. Scale bar: 20 μm. **(B)** The percentage of BrdU positive cells increased significantly after FSH treatment. **(C,D)** Western blot and gray analysis indicate that FSH promoted PCNA protein expression. **(E)** 4-day-old chicken ovaries were treated by FSH or SCF *in vitro* for 3 days. Arrowheads represent the cells with BrdU (red) marker. DAPI (blue) represents nuclear DNA. Scale bar: 30 μm. **(G)** The percentage of BrdU positive cells increased significantly with FSH and SCF treatment in combination. **(F,H)** SCF enhanced the effect of FSH to promote the PCNA protein expression. *T*-tests were used to determine statistically significant differences. The values are the mean ± SEM of six experiments. Asterisks indicate significant differences (*P < 0.05, **P < 0.01, ***P < 0.001).

**Figure 10:**
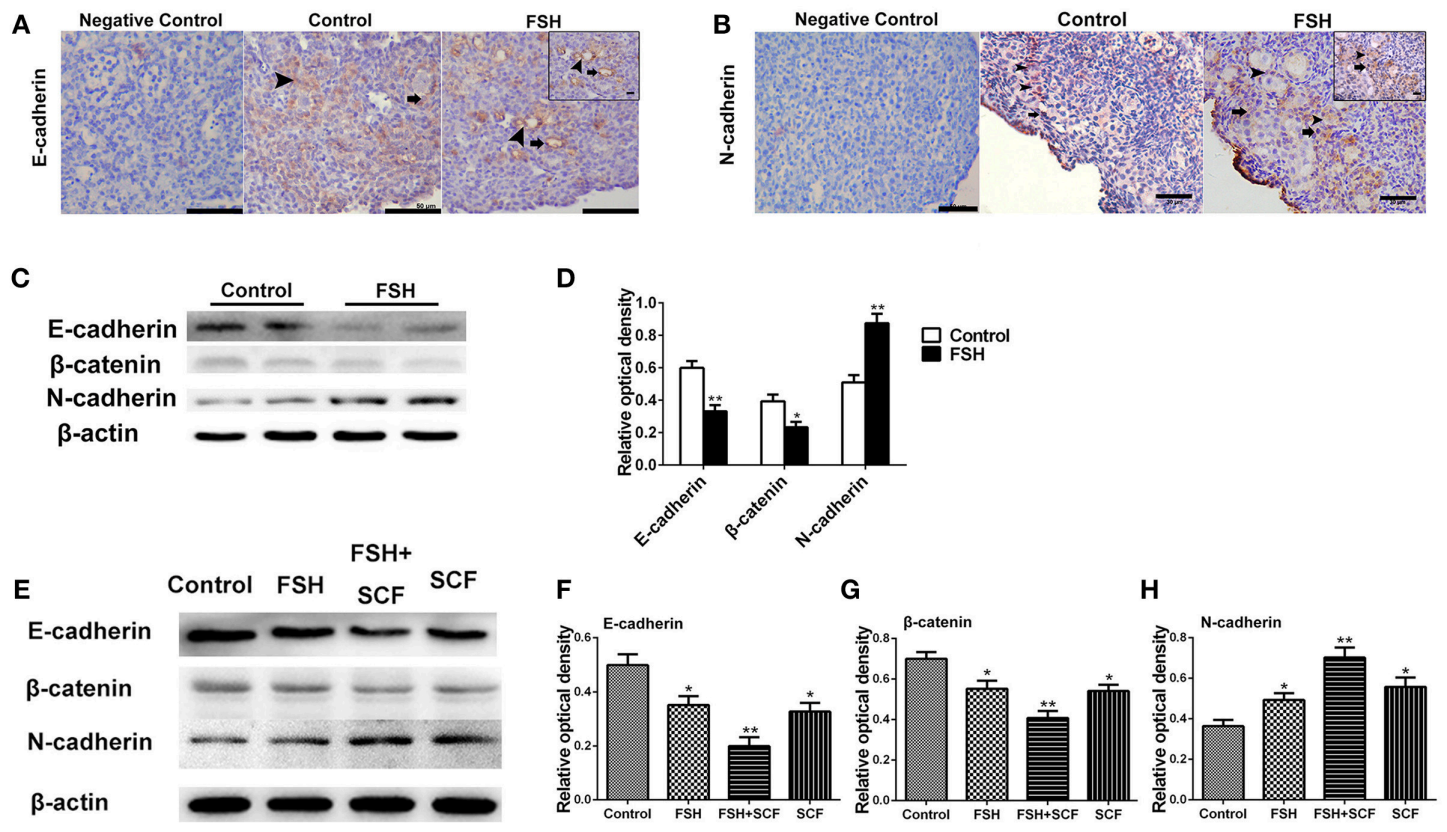
Combined effects of FSH and SCF on the expression of E-cadherin and N-cadherin. Four-day-old chickens were treated with FSH for 2 days *in vivo*. Brown stain represents immunohistochemical antigen. (**A** inset) Arrowheads and arrows represent oocyte-oocyte contact sites and somatic cells stained with E-cadherin, respectively. **(C,D)** Western blot and gray analysis indicate that FSH inhibited the E-cadherin and its partner β-catenin expression *in vivo*. (**B** inset) Arrowheads and arrows represent oocytes and somatic cells stained with N-cadherin, respectively. **(C,D)** Western blot and gray analysis indicate that FSH promoted the N-cadherin protein expression *in vivo*. **(E–H)** 4-day-old chicken ovaries were treated by FSH and SCF for 2 days *in vitro*. Western blot and gray analysis indicate that SCF enhanced the effect of FSH in promoting N-cadherin expression *in vitro*. Meanwhile, SCF augmented the inhibitory effect of FSH on E-cadherin and β-catenin expression. *T*-tests were used to determine statistically significant differences. The values are the mean ± SEM of six experiments. Asterisks indicate significant differences (*P < 0.05, **P < 0.01). Scale bars: inset in **(A,B)**, 10 μm.

With the use of immunohistochemistry and Western blot we clarified the molecular mechanism of FSH in promoting primordial follicle assembly, confirming cell proliferation, and the expression of cell adhesion molecules on the oocytes and somatic cells, respectively, after treatment with FSH for 2 days *in vivo*. Results showed that FSH treatment increased the expression of CDK2 by 65.4% (Figures 8A,C,D) and CCND1 by 78.0% (Figures 8B–D) in the somatic cells and oocytes (^*^P < 0.05). Moreover, FSH treatment increased the BrdU labeling rates by 120.0% in the ovarian cortex and significantly promoted PCNA protein expression by 42.1% in the chicken ovaries (Figures 9A–D, ^*^P < 0.05, ^**^P < 0.01). Furthermore, E-cadherin was intensely expressed at the oocyte-oocyte contacting sites inside cysts in chicken ovarian cortex. Conversely, only a few E-cadherin markers could be found in the follicular somatic cells (Figure 10A inset). FSH decreased expression of E-cadherin and its partner β-catenin by 44.6 and 40.8% (Figures 10C,D, ^*^P < 0.05, ^**^P < 0.01). Another cell adhesion molecule, N-cadherin was intensely expressed at the oocytes or somatic cells of the follicles, and weak N-cadherin expression could be detected at the oocyte-oocyte contacting sites within the germ cell cysts in the ovarian cortex. Treatment of FSH promoted the expression of N-cadherin protein by 71.6% (Figure 10B inset, c, d, ^***^P < 0.001).

### SCF Augmented the Stimulatory Effects of FSH on Primordial Follicle Formation

To confirm the interactive action of FSH and SCF to promote primordial follicle assembly, the ovarian fragments were treated with FSH and SCF *in vitro*. SCF further enhanced the promoting effect of FSH on the expressions of CDK2 and CCND1 by 45.0 and 41.4% (Figures 8E,F, ^*^P < 0.05, ^**^P < 0.01) in a reciprocal manner. Such a reciprocal effect was also found in the action of FSH on ovarian cell proliferation (represented by BrdU incorporation) and the expression of PCNA protein in the cultured ovaries which increased by 84.5 and 51.4% (Figures 9E–H, ^*^P < 0.05, ^**^P < 0.01, ^***^P < 0.001). Furthermore, treatment of the cultured ovarian fragments with FSH and SCF in combination demonstrated that SCF enhanced the inhibitory effect of FSH on the expression of E-cadherin or β-catenin protein by 43.5 and 26.2% (Figures 10E–G, ^*^P < 0.05, ^**^P < 0.01), but amplified the stimulatory effect of FSH on the expression of N-cadherin by 42.5% (Figures 1E,H, ^*^P < 0.05, ^**^P < 0.01).

**Figure 11:**
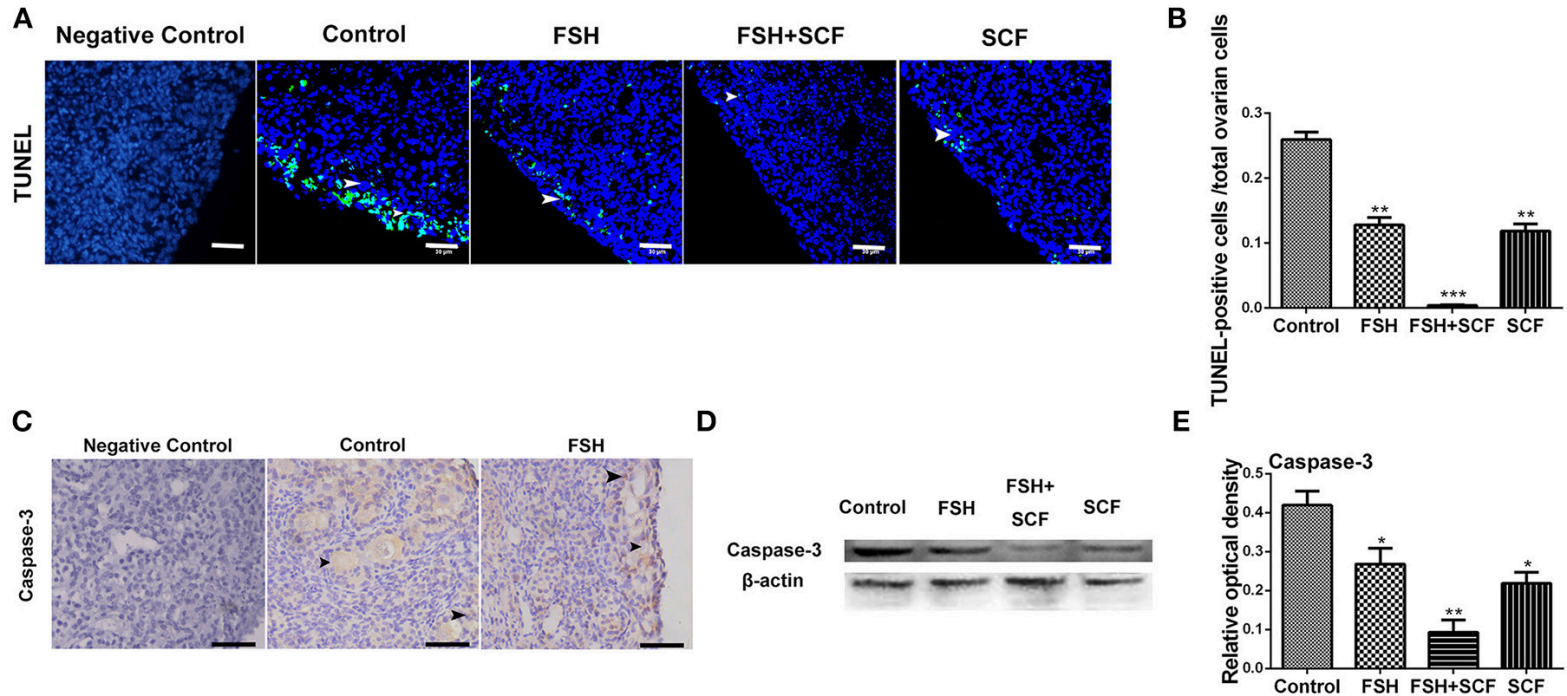
Combined effects of FSH and SCF on cell apoptosis. **(A)** Ovarian cells (arrowheads) with TUNEL (green) marker indicate cell apoptosis after the treatment of 4-day-old chicken ovaries with FSH or SCF *in vitro* for 3 days. DAPI (blue) represents nuclear DNA. Scale bar: 30 μm. **(B)** The percentage of TUNEL positive cells reduced significantly after FSH and SCF treatment. **(C)** Caspase-3 protein is indicated by a brown stain. Arrowheads represent the ovarian cells stained caspased-3 marker. **(D,E)** Western blot and gray analysis indicate that FSH inhibited caspase-3 protein expression. SCF acted to enhance the effect of FSH in inhibiting caspase-3 expression *in vitro*. *T*-tests were used to determine statistically significant differences. The values are the mean ± SEM of six experiments. Asterisks indicate significant differences (*P < 0.05, **P < 0.01, ***P < 0.001).

In addition, treatment with either FSH or SCF inhibited the apoptosis of the ovarian cells (as assessed by number of TUNEL positive cells) by 50.7 or 54.3% in cultured ovarian fragments (Figures 11A,B, ^*^P < 0.05, ^**^P < 0.01, ^***^P < 0.001). An enhancing effect of inhibiting cell apoptosis was achieved via the combined treatment of FSH and SCF. Cells positive for caspase-3 labeling (a marker of apoptosis) increased in the oocytes from the broken germ cell cysts (Figure 11C). Western blot showed that treatment of FSH and SCF together resulted in a significant effect on inhibiting the expression of caspase-3 protein by 77.8% (Figures 11D,E, ^*^P < 0.05, ^**^P < 0.01).

## Discussion

In mammals, FSH and KIT signaling are crucial for primordial follicle formation (8, 15). In the hamster, FSH has been shown to be capable of promoting primordial follicle assembly via FSHR to regulate cell adhesions and SCF production (12, 25). However, there has been little evidence to show the effects of FSH and KIT signaling on the primordial follicle assembly in avian species. The interactive effect between FSH and KIT signaling in the chicken also remains unclear. Here, results from the treatment of chickens with FSH and SCF *in vivo* and *in vitro* indicated that FSH could promote primordial follicle assembly via KIT signaling, and that SCF augmented the stimulatory effect of FSH to promote cell proliferation and the expressions of cell cycling proteins CCND1, CDK2, and E-cadherin, but also acted to inhibit cell apoptosis and N-cadherin expression in the chickens.

FSHR and SCF/c-KIT have been shown to generate positive effects on the formation of primordial follicles in mammals (15, 25). Our results examining the spatiotemporal expression of FSHR, SCF, and c-KIT indicated that they have similar time points of maximal expression, which suggests these factors in combination might be crucial for primordial follicle formation in the chicken ovarian cortex.

FSH has been noted to promote primordial follicle formation in mice and hamsters (11, 12, 22, 25, 26). By determination of the expression pattern of FSHR mRNA during early developmental stage of the chickens, we found extensive formation of the primordial follicles appeared 2 to 3 days after a sharp increase of FSHR mRNA. Treatment of the chickens with FSH *in vivo* indicated that FSH promoted the primordial follicle assembly. Our study also indicated that the stimulatory effect of FSH in promoting the primordial follicle assembly is likely to be interactive with KIT signaling. Changes of *SCF* and *c-KIT* mRNAs corresponded with the formation of the primordial follicles. Therefore, binding of SCF to its receptor c-KIT might be a reciprocal factor for FSH to promote the primordial follicle assembly after the binding of FSH to its receptor. Previous studies have revealed that AKT and MAPK signaling can regulate the formation of primordial follicles. Therefore, AKT and MAPK represents the link of FSH action to the promotion of the primordial follicle assembly. Furthermore, our results indicated FSH promoted primordial follicle assembly through KIT signaling and that SCF could increase the sensibility of ovarian cells to FSH stimulation by increasing the expression of FSHR.

Multiple hormones are known to regulate normal primordial follicle development with proper coordination of signaling pathways, transcription factors, and transposon repression (8, 27). Proliferation of the somatic cells and change in the expression of cell adhesion molecules both play important roles in primordial follicle assembly (22). In this study, we found FSH promoted primordial follicle formation by promoting ovarian cells proliferation and inhibiting cell apoptosis, acting to increase the expression of cell cycling proteins including CCND1 and CDK2 that are essential for mitosis and meiosis. CDK2 activity is essential for the first to second meiosis transition in porcine oocytes and is required for completion of prophase I of the meiotic cell cycle (28–30). Results from the current study indicate that FSH might promote primordial follicle assembly and that this occurs together with differential effects on the expressions of extracellular matrix (ECM) proteins. Therefore, the primordial follicle assembly might be accompanied with variations in cell proliferation, cell cycle, and cell adhesion in avian species.

**Figure 12:**
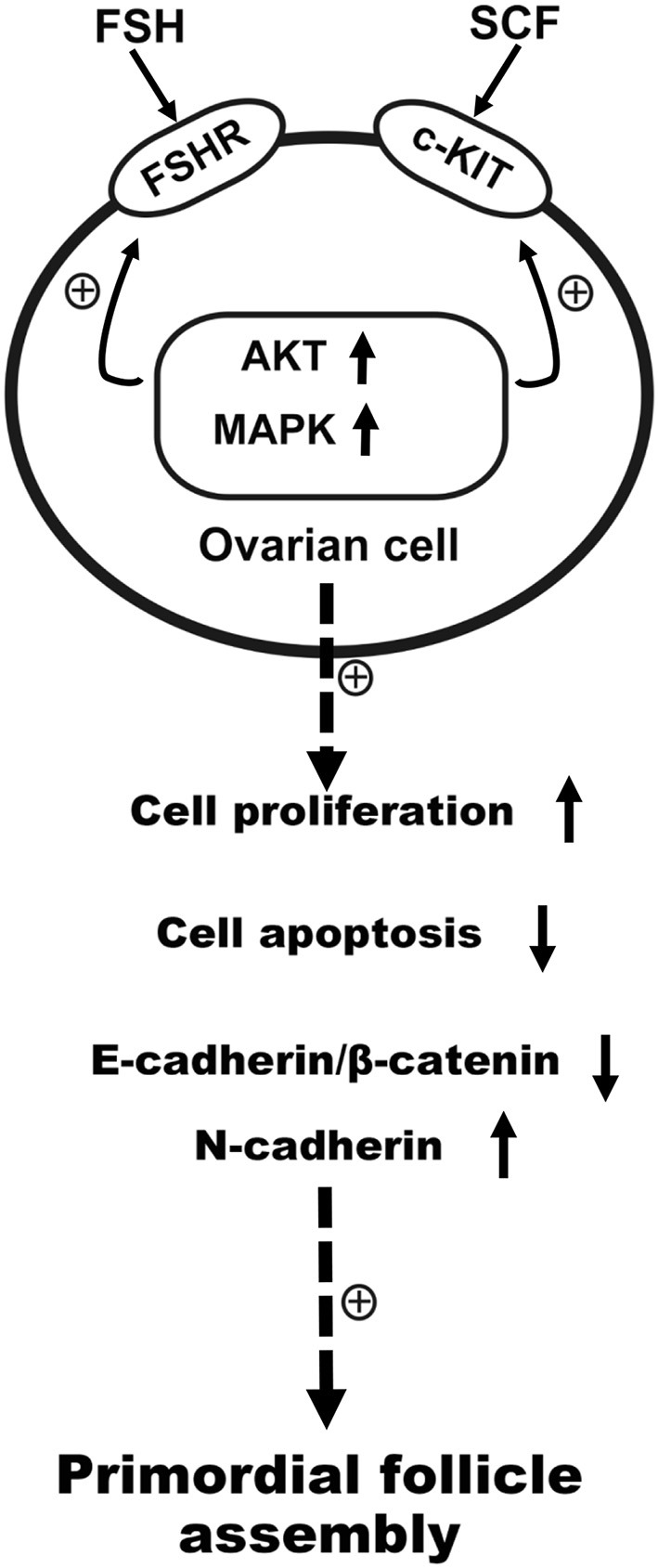
Schematic illustration of FSH and KIT signaling in promoting primordial follicle assembly in the chicken. FSH promoted primordial follicle formation, and KIT signaling augmented the sensibility of FSHR to FSH via intracellular signal transduction. In the process of primordial follicle formation in the chickens, FSH cooperated with SCF reciprocally to promote cell proliferation, the expression of N-cadherin, and transcription factors, but also acted to inhibit E-cadherin/β-catenin and cell apoptosis.

In addition to the changes in expression of cell cycle proteins, the transcription factors TAF4b and Nobox have also been noted as crucial for primordial follicle assembly (31). FSH was seen to be able to promote primordial follicle assembly via the regulation of TAF4b and Nobox (8, 31). Such studies also revealed that FSH and SCF might regulate the primordial follicle assembly in a reciprocal manner at the transcription factor level. The above results are summarized in Figure 12.

In conclusion, FSH could promote primordial follicle formation via KIT signaling. In turn KIT signaling could enhance the sensibility of FSHR to FSH by intracellular signal transduction. On the process of primordial follicle formation in chicken, FSH interacts with SCF reciprocally to accelerate cell proliferation, N-cadherin expression, and to inhibit cell apoptosis and E-cadherin expression through AKT and MAPK signaling pathway.

## Author Contributions

CG, GL, DZ, YM, CZ, and JL conceived the experiment(s). CG, GL, and DZ conducted the experiments; all authors joined the analysis and interpretation of data. CG, GL, CZ, and JL prepared the manuscript. All authors reviewed the manuscript.

### Conflict of Interest Statement

The authors declare that the research was conducted in the absence of any commercial or financial relationships that could be construed as a potential conflict of interest.
